# Antioxidant Properties and Nutritional Composition of Matcha Green Tea

**DOI:** 10.3390/foods9040483

**Published:** 2020-04-12

**Authors:** Karolina Jakubczyk, Joanna Kochman, Aleksandra Kwiatkowska, Justyna Kałduńska, Karolina Dec, Dorota Kawczuga, Katarzyna Janda

**Affiliations:** 1 Department of Human Nutrition and Metabolomics, Pomeranian Medical University, 24 Broniewskiego Street, 71-460 Szczecin, Poland; jakubczyk.kar@gmail.com (K.J.); kochmaan@gmail.com (J.K.); aleksandra.kwiatkowska.96@gmail.com (A.K.); justynakaldunska@wp.pl (J.K.); karolina_dec@wp.pl (K.D.); 2 Institute of Biology, University of Szczecin, 3c Felczaka Street, 71-412 Szczecin, Poland; dorota.kawczuga@usz.edu.pl

**Keywords:** Matcha, antioxidant, polyphenols, rutin, vitamin C, green tea

## Abstract

Matcha green tea (*Camellia sinensis*), which originates from Japan, is commonly considered as particularly beneficial to health. A large content of polyphenols, amino acids (mainly tannins) and caffeine potentially increase the antioxidant properties of the drink. The aim of the study was to determine the antioxidant potential and the content of substances with an antioxidant effect—vitamin C, total polyphenol content including flavonoids—in infusions made from Traditional Matcha (from the first and second harvests) and Daily Matcha (from the second and third harvests) at different temperatures. The infusions were made by pouring 100 mL of distilled water once at various temperatures (25 °C, 70 °C, 80 °C and 90 °C) over 1.75 g of the plant material. Matcha tea is characterized by a high level of antioxidant substances (flavonoids 1968.8 mg/L; polyphenols 1765.1 mg/L; vitamin C 44.8 mg/L) as well as antioxidant potential (41.2% DPPH (10× dilution); 6129.5 µM Fe(II)/dm^3^ FRAP). The concentration of these compounds depends on the time at which the plant material was harvested as well as on the temperature of water used to prepare the infusions. For most parameters, the highest values were observed in infusions prepared at 90 °C and from the daily Matcha.

## 1. Introduction

During stressful situations—including environmental pollution, improper diet, chronic psychological stress, very intense and prolonged physical exertion, starvation diets leading to significant malnutrition, broadly understood infections—the cells of the human body are susceptible to the excessive synthesis of reactive oxygen species [1,2]. During the domination of oxidative stress over defensive mechanisms, cells suffer damage to their genetic material, cell membrane and building proteins, which leads to numerous disorders [3]. Serious consequences include illnesses with a free-radical basis, such as diabetes, atherosclerosis, neoplastic disease, neurodegenerative diseases or the aging of the organism [4,5,6]. One solution to maintaining the appropriate oxidative balance of the organism is a high supply of exogenic antioxidants with the aim of equalizing and preventing oxidative processes, and it is also important to maintain a correct lifestyle, free from stress-inducing factors [7].

Tea is second to water in terms of the frequency of liquid consumption in the world [8]. It is valued for its characteristic taste and aroma, beneficial health values and social-cultural conditions [9,10]. Green tea is available for sale in three variants: In the form of leaves, in tea bags or as powder [11]. Matcha is a powdered type of Japanese green tea (*Camellia sinensis*) of the Tencha type [12], commonly consumed in the world, and the frequency of its consumption keeps increasing [13]. This tea type is particularly rich in antioxidant compounds due to its traditional way of cultivation [14,15]. Ideally, during most of its growth time [16] it is shaded by natural bamboo fabric [11]. This method of protection against sun allows the plant to create high amounts of bioactive compounds, including chlorophyll and l-theanine. The results of cultivating the plant in shade before harvest are the unique taste and the color of the resource and its infusions, but the value and content of the tea also depend on the season when the leaves are harvested [17,18]. The high content of theanine and caffeine and low content of catechin result in higher amounts of ingredients providing the “umami” taste in comparison to other tea types cultivated in full sun, in which catechin content amplifies bitter taste [18]. This is why Matcha is considered as the most aromatic green tea and a product of the highest quality [12,16].

The proven benefits of green tea result from the presence of antioxidants, such as polyphenols which include a large number of compounds: Flavonols, flavandiols and phenolic acids, constituting even up to 30% of dry mass [9,19]. Due to the antioxidant, antiviral and anti-inflammatory function, stimulating immunological and detoxification processes, the consumption of green tea is associated with a reduced risk of circulatory system illnesses and cancers by delaying the occurrence of factors associated with the progression of those illnesses [20,21,22]. The content of healthy constituents of teas depends on the type of tea, its quantity, temperature and time of brewing [23]. Despite its long tradition, Matcha tea appeared on the market relatively recently and there are not many studies on this resource. This is why the aim of this research was to label the antioxidant potential and the content of substances with an antioxidant effect in various types of Matcha tea in order to support the regular consumption of this drink as a potential method for preventing illnesses, including those with a free-radical basis.

## 2. Materials and Methods

### 2.1. Plant Material

The studied material consisted of two types of high quality organic Japanese powder, green Matcha tea (*Camellia sinensis*), made from the leaves of Tencha, originating from the Uji region of Japan in the Kyoto prefecture. Traditional Matcha came from the first and second harvest of the leaves, whereas Daily Matcha came from the second and third harvest.

### 2.2. Preparation of Infusion

1.75 g of a plant material sample was transferred to a conical flask to which 100.0 mL of distilled water was added at a given temperature (25 °C, 70 °C, 80 °C, and 90 °C, most commonly used to prepare plant infusions). The flask with the infusion was closed and rotated with a speed of 180 rpm (Brunswick model EXCELLA E24) for 10 min. After brewing, the plant parts were separated from the infusion by filtration. The infusions were cooled to room temperature and analyses were carried out immediately. All the infusions were repeated three times.

### 2.3. Antioxidant Activity of Infusion by the DPPH Method

The antioxidant activity of samples was measured with the spectrophotometric method, using synthetic radical DPPH (2.2-diphenyl-1-picrylhydrazyl, Sigma, Poznań, Poland) according to Brand-Williams et al. and Pekkarinen et al. [24,25]. The samples were diluted 10 times. The spectral absorbance was immediately measured at 518 nm (Agilent 8453UV). The results were expressed as % inhibition of DPPH radical. All assays were performed in triplicate.

### 2.4. Determination of Reduction Potential of Infusion by the Ferric Ion Reducing Antioxidant Power (FRAP) Method

The FRAP method, used to determine the total reduction potential, is based on the ability of the test sample to reduce Fe^3+^ ions to Fe^2+^ ions. The FRAP unit determines the ability to reduce 1 mole Fe^3+^ to Fe^2+^ [26,27]. Absorbance at 593 nm was measured (Agilent 8453UV). The results were expressed as µM Fe(II)/dm^3^. All assays were performed in triplicate. 

### 2.5. Determination of the Total Polyphenols Content (TPC) in Infusion

Determination of the total polyphenols content (TPC) was performed according to ISO 14502-1 and Singleton V.L., Rossi J.A method using Folin–Ciocalteu reagent [28]. Absorbance at 765 nm was measured (Agilent 8453UV). All assays were performed in triplicate. The results are shown in mg/L gallic acid.

### 2.6. Determination of the Total Flavonoids Content (TFC)

Determination of total flavonoids content was performed according to the methods in Hu et al. [29]. Different concentrations of rutin were used in the plotting of the standard calibration curve. The TFC was expressed as mg of rutin equivalent per 1 L of fresh Matcha tea. Absorbance at 510 nm was measured (Agilent 8453UV). All assays were performed in triplicate.

### 2.7. Determination of the Content of Vitamin C

The quantitative labeling of the content of ascorbic acid in the infusions was performed according to Tillmans et al.’s method [30]. Titration is based on the reaction proceeding along with the change in the color of the solution of ascorbic acid under the influence of the solution of 2.6-dichloroindophenol. The samples were titrated until light-pink coloring was achieved, which lasted for 10 s. The result was presented in mg of ascorbic acid per 1 L of the infusion. Each labeling was performed three times.

### 2.8. Statistical Analysis

In all the experiments, three individual infusion samples were analyzed and all the analyses were carried out at least in triplicate (9 replications).

The statistical analysis was performed using Stat Soft Statistica 13.0 and Microsoft Excel 2017. The results are expressed as mean values ± standard deviation (SD).

To assess the differences between examined parameters one-way analysis of variance (ANOVA) with Tukey’s post-hoc test was used. Differences were considered significant at *p* ≤ 0.05.

## 3. Results

The results reported in this article show how Matcha infusions (Daily, Traditional) affect the total content of polyphenols, rutin, vitamin C and the antioxidant properties. Matcha tea infusions have high antioxidant potential. The values of antioxidant potential of the studied infusions, expressed as the reductive values of iron ions, were in the range between 5767.30 and 6129.53 µM Fe(II)/dm^3^. Higher values were observed for Traditional Matcha (5863.03–6129.53 µM Fe(II)/dm^3^), whereas for Daily Matcha the values were lower (5767.30–5896.95 µM Fe(II)/dm^3^). Statistically significant differences between Traditional Matcha and Daily Matcha were observed for all temperatures except 25 °C (*p* < 0.05) (Table 1). Furthermore, a rising tendency was observed based on temperature. In both cases, the lowest potential was observed at 25 °C, and the highest at 90 °C. For Traditional Matcha, statistically significant differences were observed between 90 °C and the other variants (*p* < 0.05), whereas for Daily Matcha, the differences occurred between 25 °C and 80/90 °C (*p* = 0.000175, *p* = 0.000176, respectively) and between 70 °C and 80/90 °C (*p* = 0.000175, *p* = 0.000184, respectively) (Table 1).

The values of antioxidant potential of the studied infusions, presented as the percentage of the inhibition of the DPPH radical, were between 12.08 and 41.24% DPPH inhibition for 10× diluted infusions. Daily Matcha was characterized by higher antioxidant potential than Traditional Matcha (33.60–41.24% in comparison to 12.08–23.48%). For all temperatures, statistically significant differences were observed between the two tea types (*p* < 0.05) (Table 1). In this case, there was also an increasing tendency depending on temperature. For both Matcha types, the lowest potential was observed at 25 °C, whereas the highest was at 90 °C (Table 1). For both tea types, statistically significant differences were observed between 90 °C and the other variants. Additionally, in the case of Traditional Matcha, statistically significant differences were observed for 25 °C vs 80 °C (*p* = 0.00386) (Table 1).

In the present study, it has been shown that the content of polyphenols in the infusions was similar and ranged between 1345.41 and 1577.01 mg/L (at 25 °C and 90 °C, respectively) for Traditional Matcha, and between 1620.63 and 1765.12 mg/L for Daily Matcha (at 25 °C and 90 °C, respectively) (Table 2). The highest content of polyphenols in Daily Matcha (1765.12 mg/L) was demonstrated in the infusion made at 90 °C, and the lowest one appeared when the infusion was made at 25 °C (1620.63 mg/L). The range of total content of polyphenols was lower in Traditional Matcha than the Daily Matcha infusion. The highest content of polyphenols (1577.01 mg/L) was demonstrated in the infusion made at 80 °C, and the lowest one appeared when the infusion was made at 25 °C (1345.41 mg/L) in Traditional Matcha infusion. Significant differences were observed between the infusion prepared at 25 °C vs 80 °C for the Traditional infusion (*p* = 0.0300) and 25 °C vs 90 °C for the Daily variant (*p* = 0.0214) (Table 2).

Matcha infusions seem to be a good source of vitamin C. The average content of this vitamin in the infusions made from Traditional Matcha was from 32.12 to 41.06 mg/L, whereas for Daily Matcha, the range was 34.9–44.8 mg/L. The highest concentration was observed at 25 °C and 70 °C, whereas the lowest was at 90 °C. Statistically significant differences both for Daily and Traditional Matcha were observed for all temperatures (*p* < 0.05) with a few exceptions. For Traditional Matcha, the differences in content of vitamin C were visible between 80 °C and 90 °C (*p* = 0.9983), whereas for Daily Matcha, it was between 25 °C and 70 °C (*p* = 0.9609) and between 80 °C and 90 °C (*p* = 0.7647) (Table 2).

The infusions were also characterized by a high content of flavonoids. A higher concentration of this compound was observed in the Daily Matcha originating from the second and third harvest. For all temperatures, statistically significant differences were observed between Traditional Matcha and Daily Matcha (*p* < 0.05) (Table 2). A similar tendency was observed when measuring polyphenol compounds. The concentration of flavonoids in Daily Matcha was between 1379.82 and 1968.79 mg/L, whereas in Traditional Matcha the range was 1222.6–1514.28 mg/L. Statistically significant differences for Daily Matcha were observed between 90 °C and 25 °C, 70 °C, 80 °C (*p* = 0.4671, *p* = 0.0115, *p* = 0.0121, respectively). For Traditional Matcha, the differences were observed between 70 °C and 25 °C, 80 °C (*p* = 0.0004, *p* = 0.0204, respectively), and between 90 °C and 25 °C, 80 °C (*p* = 0.0002, *p* = 0.0041) (Table 2).

## 4. Discussion

The hazards associated with excessive accumulation of free radicals can be prevented not only by the internal antioxidant system, but also by antioxidant substances consumed with foods, particularly those of plant origin [31]. Plant resources are a rich source of antioxidant compounds which, according to their definition, are characterized by delaying or inhibiting oxidative processes, protecting the organism against the effects of free radicals [32]. Despite its increasing popularity, our knowledge of Matcha is still largely vague and the name of the tea itself is often replaced by the broader term, green tea. In this study, we analyzed the content of polyphenols, flavonoids, vitamin C as well as the antioxidant potential of Matcha collected at different times and used in infusions at different temperatures.

Polyphenol compounds are considered as strong antioxidants. They have a stronger antioxidant effect compared to vitamins, such as vitamin C and E, and they include carotene and tocopherol, which have the potential to alleviate oxidative stress [33,34,35]. Apart from antioxidant mechanisms, polyphenols also have the ability to uptake metabolically active carcinogens and to prevent the mutagenicity and genotoxicity of compounds, which contribute to their strong antineoplastic properties [36,37]. High content of these compounds was observed in this study. The content of polyphenol compounds in our study was between 1345.41 and 1765.12 mg/L. The highest content of polyphenols was observed when the tea was brewed at 90 °C for 10 min, which was similar to the result achieved by other researchers who observed that the highest content of bioactive compounds of tea infusions was at 80 °C during a relatively short brewing time (5–10 min) [9,38]. These results point to the fact that short brewing time in combination with high brewing temperature of water yield the best results when extracting tea compounds [39]. Komes et al. [9] analyzed the content of polyphenol compounds in various forms (leaves, tea bags, powder) of green teas. Matcha, brewed at the highest temperature (100 °C), was characterized by one of the highest concentrations of polyphenols (2230 mg/L) [9]. However, it is worth pointing out that the polyphenols in our study showed to be more stable than in Komes et al.’s [9] study, and temperature did not have a significant influence on their concentration. Other studies have noted that using lower brewing temperatures but longer extraction times for plant material can increase antioxidant activity by protecting specific polyphenolic fractions [40,41].

Matcha is one of the richer sources of flavonoids, especially rutin. Rutin is a compound that has an antioxidant effect and belongs to the group of polyphenols. Additionally, it helps to seal blood vessels, has anti-inflammatory properties and it also supports the immune system [42]. Furthermore, it also slows down the oxidation of vitamin C, which results in numerous supplements available on the market that contain a combination of the two constituents. The content of rutin equivalent in Matcha is between 1222.6 and 1968.8 mg/L. Daily Matcha seems to be a slightly better source of flavonoids (rutin equivalent). In comparison, buckwheat—considered as one of the best sources of rutin—contains 62.30 mg/100 g of fresh weight. The tea drink available on the market included only 19.68 mg/100 mL of the infusion [43]. Out of 14 of the studied infusions from various tea types, green tea included the highest content of rutin—37.13 mg/L—nearly 50 times less than Matcha. Therefore, it should be highlighted that Matcha, as a drink, is the best source of rutin, and in comparison to all plant resources, it occupies a very high position. Additionally, the infusions also contain vitamin C, which is particularly important when the content of rutin is so high [44]. Through the synergistic effect of rutin and vitamin C, Matcha also has an influence on the circulatory system. Vitamin C also has an influence on the synthesis of collagen, which is the main structure-forming protein in the human body [45]. A resource that is rich both in rutin and vitamin C can successfully support the circulatory system and protect against oxidative stress.

Studies that confirm the high antioxidant potential of tea beverages point to catechins as the significant source of this potential. Catechins belong to the group of polyphenols, which have a beneficial effect on human health, and occur naturally in the leaves of *Camelia sinensis*. Other studies also support the notion that the tea can be considered as the main source of catechins in everyday human diets [8,46].

Epigallocatechin gallate (EGCG), epigallocatechin (EGC), epicatechin gallate (ECG) and epicatechin are the main active substances of the catechin type and are present in the highest amount in plant resources. Catechins of tea origin are characterized by particularly strong antioxidant activity due to their ability to neutralize free radicals and to increase the detoxification activity of enzymes, which include (among others) glutathione peroxidase, catalase reductase and glutathione reductase. The properties mentioned above may be reflected in the potentially preventive effect on atherosclerosis, as well as on the increase in the elasticity of arteries, indirectly preventing ischemic heart disease [15,47]. Catechins, by protecting cells against the activity of reactive oxygen species which can lead to the destruction of proteins, lipids and DNA, prevent the premature aging of the cells of the organism and the brain [8]. Through the systematic and long-term consumption of tea beverages the development of illnesses with a free-radical base, including neurodegenerative diseases, is also delayed. In particular, strong neuroprotective effects have been observed for the main component of catechins, which is epigallocatechin gallate [48]. Additionally, EGCG may also prevent inflammations occurring in organisms via the inhibition of the creation of proinflammatory particles by the immune system [15].

Matcha is characterized by high antioxidant potential. The value for the antioxidant status of the studied infusions, expressed as the percentage of DPPH radical inhibition, was in the range between 12.07% and 41.24%. However, it has to be highlighted that these infusions were 10× diluted. The antioxidant potential for the FRAP method was in the range between 5767.30 and 6129.53 µM Fe(II)/dm^3^. Our study is consistent with the results achieved by other authors, who confirmed the highest antioxidant potential for the infusions. The antioxidant potential of green tea infusions was in the range of 60–80% of DPPH inhibition [49]. Compared to other green teas (leaves, bags), Matcha tea is characterized by the highest antioxidant potential (FRAP, DPPH) [9,11].

Antioxidant potential, measured using FRAP for Daily Matcha, turned out to be higher in comparison to Traditional Matcha, but the DPPH method showed a reverse correlation. The differences stem from the limitations and the capabilities of both methods. The FRAP method is based on the measurement of the reductive ability of the substance through the reduction of iron ions Fe^3+^ into Fe^2+^, the donor ability of the antioxidant [46]. However, when using the FRAP method, compounds such as thiols, glutathione and proteins do not show reductive properties in relation to iron (III). Additionally, the quality limiting this method is the dependence of the durability of this complex on the pH of the solution, which is 3.6 in optimal conditions. Another limitation is the non-selectivity in relation to the reduction of the iron (III) complex with (2.4.6-tris(2-pyridyl)-1.3.5-triazine), not only through the desired antioxidant compounds [50]. The test with the DPPH radical aims at labeling the ability of its neutralization via the antioxidants included in the solution [51]. The limiting parameter in the DPPH method is the lack of the ability to label hydrophilic antioxidants due to the fact that DPPH is soluble only in organic solvents, mainly alcohol [52]. When performing the labeling, the studied sample needs to be protected against oxygen and light, and it is also important to correctly adjust pH and its solvent, not to decrease the absorbance of the DPPH solution [53]. This is why the results of this method can be lower, which is visible in our studies in reference to Traditional Matcha. In our study, we used both of the aforementioned methods of labeling to achieve a wide spectrum of results and an overall image of the constituents of the antioxidant potential of Matcha green tea, eliminating possible interference and limiting factors.

An important parameter that has an influence on the acquired results is the temperature of water used to prepare the infusions. In the case of both methods, the antioxidant potential was higher at 90 °C, and the lowest at 25 °C. This is associated with the better release of biologically active compounds and the higher kinetic energy at higher brewing temperatures, as was also indicated in similar studies [9,54].

Our observations indicate that Traditional Matcha originating from the first and second harvest includes less polyphenols, including rutin and vitamin C, and is characterized by lower antioxidant potential (DPPH) than Daily Matcha originating from the second and third harvests. Statistically significant differences were observed in reference to all of the studied parameters and at all of the studied temperatures (*p* < 0.05). The only exception was the antioxidant potential measured via the FRAP method at 25 °C. The results of this study are similar to those achieved by other authors. Ku et al. [18] confirmed the differences between April and July harvests. The April harvest was characterized by lower antioxidant potential in contrast to the July harvest. The differences in the results between the harvests may stem from the fact that phytochemical content of tea is influenced by factors such as: The conditions of cultivation, the age of the leaves, storage or even the type of processing [35,55]. The shading of the leaves also has an influence on the reduced production of catechins, which is reflected in the content of polyphenols in the infusion [18]. Furthermore, leaf collection during the first harvest may be associated with an increased synthesis of secondary metabolites of the plant. Therefore, it can be concluded that leaves collected during the second and third harvests are characterized by higher compound content and more beneficial qualities for the human health than the ones collected during the first harvest.

Matcha’s high antioxidant potential is also visible in its powdered form. Fujioka et al. [54] proved that powdered tea, in comparison to leaf tea, is characterized by a higher concentration of polyphenols when using the same amount of leaves and powder. This suggests that the grinding process itself has an influence on the acceleration of polyphenol extraction and the mixing process during brewing. Shishikura and Khokhar [56] also observed that when taking into account the average time of brewing the tea, its powdered form is a better choice as it is more effective and active in terms of the extraction and takes less time (1 min). In a study conducted by Komes et al. [9], 11 types of green tea were analyzed in various forms (tea bags, leaves, and powdered, Matcha), brewed at 60 °C, 80 °C and 100 °C and subjected to various brewing times 3, 5, 10, 15 and 30 min. The influence of grinded raw material as well as the time and temperature of the infusions on the content of phenolic compounds and their antioxidant capabilities (DPPH-2.2-diphenyl-1-picrylhydrazyl, FRAP -Ferric ion reducing antioxidant power, ABTS-2,2′-azino-bis(3-ethylbenzothiazoline-6-sulfonic acid) were analyzed. In all green tea types, there was an increase in the antioxidant potential with the increase in water temperature, and the highest value for each tea type was achieved at 100 °C and the brewing time of 3 min. It has been concluded that the potential of green tea increases with phenol content. Additionally, the powdered form (Matcha) was characterized by the highest parameters out of all green tea types and required the shortest time of brewing. The increase of extraction time of the powdered Matcha did not have an influence on its antioxidant properties [11].

## 5. Conclusions

Matcha, the Japanese powdered green tea, is a valuable resource with rich content of biologically active substances with antioxidant properties. It is characterized by particularly high content of rutin, but also polyphenols and vitamin C. Additionally, its infusions have a high antioxidant potential, the highest out of all tea types. However, factors such as the time of harvest and the temperature of brewing have an influence on its properties. Infusions made from Matcha tea, particularly from the second and third harvests, can be a valuable source of antioxidants and can be used in the prophylaxis of illnesses with a free-radical basis.

## Figures and Tables

**Table 1:** Antioxidant potential (DPPH, FRAP) in Matcha tea infusion (Traditional, Daily).

Type of Matcha	Temperature	DPPH	FRAP
	°C	%	µM Fe(II)/dm^3^
**Traditional-MT**	25 ^a^	12.08 ± 0.58 ^*c,d,e^	5863.03 ± 156.51 ^*b,c, d^
	70 ^b^	14.40 ± 2.65 ^*d,f^	6062.79 ± 33.93 ^*a,f^
	80 ^c^	18.11 ± 3.13 ^*a,d,g^	6088.57 ± 133.22 ^*a, g^
	90 ^d^	23.48 ± 5.54 ^*a,b,c,h^	6129.53 ± 68.40 ^*a,h^
**Daily-MD**	25 ^e^	33.60 ± 0.21 ^*f,g,h,a^	5767.23±16.13 ^*g,h^
	70 ^f^	39.05 ± 2.45 ^*e,b^	5791.80 ± 41.91 ^*g,h,b^
	80 ^g^	37.26 ± 1.53 ^* e,c^	5923.10 ± 41.94 ^*e,f,c^
	90 ^h^	41.24 ± 0.84 ^*e,d^	5896.95 ± 16.10 ^*e,f,d^

*p ≤ 0.05 between particular subgroup: ^a^—MT, 25 °C; ^b^—MT, 70 °C; ^c^—MT, 80 °C; ^d^—MT, 90 °C; ^e^—MD, 25 °C; ^f^—MD, 70 °C; ^g^—MD, 80 °C; ^h^—MD, 90 °C. MT—Traditional Matcha, MD—Daily Matcha

**Table 2:** The total polyphenols content (TPC), flavonoids and vitamin C in Matcha tea infusion (Traditional, Daily).

Type of Matcha	Temperature	TPC	Flavonoids	Vitamin C
	[°C]	[mg/L]	[mg/L]	[mg/L]
**Traditional-MT**	25 ^a^	1345.41 ± 238.69 ^*c,e^	1222.60 ± 14.94 ^*b,d,e^	41.06 ± 0.11 ^*b,c,d,e^
	70 ^b^	1499.88 ± 73.03 ^*f^	1514.28 ± 20.37 ^*a,c,f^	35.48 ± 0.07 ^*a,c,d,f^
	80 ^c^	1577.01 ± 43.22 ^*a,g^	1340.77 ± 118.54 ^*b,d,g^	32.25 ± 0.06 ^*a,b,g^
	90 ^d^	1495.47 ± 150.33 ^*h^	1460.41 ± 202.77 ^*a,c,h^	32.12 ± 0.10 ^*a,b,h^
**Daily-MD**	25 ^e^	1620.63 ± 50.09 ^*h,a^	1723.11 ± 108.78 ^*h,a^	44.30 ± 0.14 ^*g,h,a^
	70 ^f^	1736.31 ± 143.22 ^*b^	1802.44 ± 154.90 ^*h,b^	44.80 ± 0.24 ^*g,h,b^
	80 ^g^	1698.32 ± 58.57 ^*c^	1968.79 ± 154.35 ^*h,c^	34.90 ± 0.19 ^*e,f,c^
	90 ^h^	1765.12 ± 99.11 ^*e,d^	1379.82 ± 101.57 ^*e,f,g,d^	36.00 ± 0.30 ^*e,f,d^

*p**≤** 0.05 between particular subgroup: ^a^—MT, 25 °C; ^b^—MT, 70 °C; ^c^—MT, 80 °C; ^d^—MT, 90 °C; ^e^—MD, 25 °C; ^f^—MD, 70 °C; ^g^—MD, 80 °C; ^h^—MD, 90 °C.
